# EHMTI-0234. Glyceryl trinitrate provoked mast cell degranulation is secondary to the release of nitric oxide in vivo

**DOI:** 10.1186/1129-2377-15-S1-F10

**Published:** 2014-09-18

**Authors:** S Hougaard Pedersen, R Ramachandran, DV Amrutkar, S Petersen, J Olesen, I Jansen-Olesen

**Affiliations:** 1 Glostrup Research Institute, Danish Headache Center, Glostrup, Denmark; 2 Department of Neurology, Danish Headache Center, Glostrup, Denmark

## Introduction

Infusion of the nitric oxide (NO) donor, glyceryl trinitrate (GTN), provokes a migraine-like attack in migraineurs and cFos up-regulation in rat trigeminal nucleus caudalis. We studied the level of dura mast cell degranulation after infusion of GTN in awake, freely moving rats and hypothesized that the degranulation is secondary to NO.

## Aims

We studied the effect on dura mast cell degranulation of NO from GTN in vivo and of GTN and sodium nitroprusside (SNP) ex vivo.

## Methods

GTN was infused i.v. in rats and mast cell degranulation was evaluated at different time points (Approved by Animal Experiments Inspectorate). Dura mater was subjected to GTN and SNP ex vivo and mast cell degranulation was likewise evaluated. Release of NO was confirmed by cerebral artery relaxation in a wire myograph setup.

## Results

GTN infusion in vivo induced a significant mast cell degranulation in a time-dependent manner. Degranulation increased from 30 min (20.3 ±3.0%) to 2 hours (61.0 ±15.3%) reaching a stable level for at least 6 hours (54.5 ±9.3%). In an ex vivo setup, neither of the NO donors GTN (9.65 ±1.17%) or SNP (11.63 ±3.46%) caused mast cell degranulation compared to vehicle (11.65 ±2.89% and 9.97 ±3.09%, respectively) after 10 min. Cerebral arteries dilated significantly in response to both GTN (68.0 ±1.0%) and SNP (73.5 ±2.5%).

## Conclusions

In vivo infusion of GTN caused significant mast cell degranulation 30 min to at least 6 hrs after infusion, suggesting a secondary effect of NO. In support of this, direct application of NO did not degranulate dura mast cells ex vivo.

No conflict of interest.

